# Prof. Hotz’ Operation for Trichiasis

**Published:** 1888-01

**Authors:** F. E. Yoakum

**Affiliations:** Chicago, Illinois


### CORRESPONDENCE.

### A LETTER FROM DR. YOAKUM.

DR. HOTZ'S IMPROVED OPERATION FOR TRICHIASIS.

CHICAGO, ILLINOIS, January 1, 1888.

*Editor Daniel's Texas Medical Journal.*

DEAR SIR:—According to promise I write you a letter for publication, and I have decided to describe Dr. Hotz's Improved Operation for Trichiasis. (Dr. Hotz is professor of Operative Surgery of the Eye in the Illinois Eye and Ear Infirmary of Chicago.)

This operation is a modification of the one Dr. Hotz described in the Archives of Ophthalmology in 1879, and as now modified, was reported at the last International Congress. This operation has for its chief point the restoration of the ciliary border to its normal position. I have witnessed many operations for this common trouble; and last winter, when I saw the tarsal cartilage grooved at its upper border, and the skinflap securely fastened into this grooved cartilage, I thought it was *the operation*. But when I saw in my practice the extreme cases of trichiasis, the turning in of the ciliary border, I did not believe this operation would accomplish the desired result in all cases.

When I see in this operation of Dr. Hotz, the restoration of the ciliary border of the lids to its natural position, I believe it to be preferable to all the other operations for this common disease.

OPERATION

as I saw it performed by the doctor, December 15, 1887.

Patient, male; age, 50; had been operated on in the old way three times before, without any benefit whatever, only making this operation more tedious. The patient being fully etherized, (for they use no chloroform in this institution,) the upper lid tissues were made tense by forceps holding the free border, and the thumb of the assistant pressing on the supra orbital ridge, where an incision was made with a sharp scalpel, across the lid, from one side to the other, down through all the tissues boldly to the cartilage, just below the superior border of cartilage. And this border is easily located by the external furrow that is formed by the folding of the upper lid tissues. (You can see this external line, or furrow, by pulling down any person's upper lid.) Then carefully dissecting up the lower flap of skin together with all the muscular tissues ; this dissection is carried down to the bulbs of eyelashes. Then a transverse incision is made through the tarsal cartilage, just above the hair bulbs, and is made in such a direction that if carried on beyond the cartilage, would just sever the free border from the conjunctiva; and also another incision is made parallel with the first one through the cartilage, from nasal side to outer canthus. Instead of the last incision being carried directly through the cartilage, it is carried slanting toward the lower. incision, making when dissected out, a V shaped groove or furrow. These incisions are about two m. m, apart on outer side of cartilage. When the bleeding has been checked, the flaps of skin are brought together by passing a needle, armed with a No. 2 silk thread through the superior border of lower flap, thence through the superior border of tarsal cartilage, thence through the inferior border of upper flap. About four of these sutures are sufficient for one lid. When these are carefully brought together and tied, the ciliary border of lid is turned up and stands out naturally, and the eyelashes, of course, are then turned up and removed from the cornea ; thereby removing the irritation that has been going on for years. One can see that it is almost impossible for the lashes to curve in again on the eyeball.

We meet patients every day who have had many operations made on their lids, and refuse to have any others performed, for they say, in a few days, or weeks, they will be worse than ever, for the old operation makes this disease more complicated ;—then why should we cóntinue such practice that really does more harm than good ?

I write this letter hoping that surgeons will study up this matter, and do this class of sufferers permanent good when operated upon.

Fraternally,

F. E. YOAKUM, M. D.

P. S. I was kindly invited into the parlor of Dr. Hotz the other day to look at some fine paintings, and while examining those beautiful scenes, life-like and grand, my eye was attracted to a bust figure in one corner of the parlor, life-size, carved out of the whitest marble. Asking "Doctor, who is that you prize so highly?" his answer was: "That is the man with whom I studied the eye, the great von Graefe." This bust brought to my mind "our own" Dr. Cupples, for they look as much alike as if they were brothers. [Just whisper this to Dr. Cupples, please.]

Yours truly,

